# Supplementary material for: ACID: A comprehensive toolbox for image processing and modeling of brain, spinal cord, and ex vivo diffusion MRI data
Source: Imaging Neurosci (Camb). 2024 Sep 13;2:imag-2-00288. doi: 10.1162/imag_a_00288 (PMC12290833; doi:10.1162/imag_a_00288)
Supplement: Supplementary Material [file imag_a_00288-supp.pdf]

Supplementary material

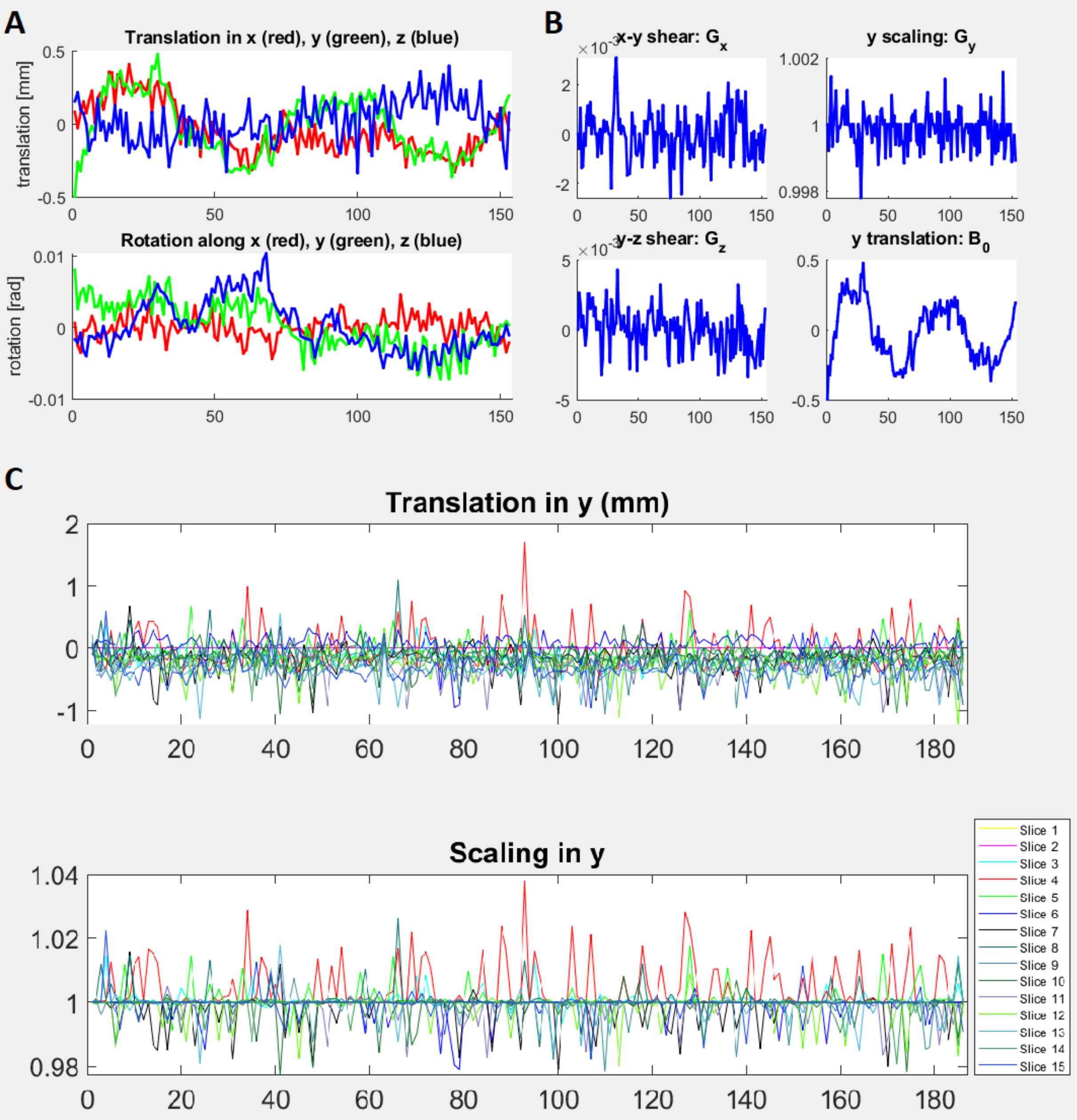

Fig. S1. Diagnostic plots, optionally generated by ECMOCO, displaying the transformation parameters for all volumes (in case of volume-wise registration) or slices (in case of slice-wise registration). For volume-wise registration, demonstrated here with an in vivo brain dMRI dataset, two figures are created to plot the transformation parameters associated with motion (A) and eddy-current-related displacements (B). For slice-wise registration, shown here with an in vivo spinal cord dMRI dataset, a single figure is created to plot the transformation parameters with separate subfigures for each estimated degree of freedom (C). Excessive displacements in volumes/slices indicate extreme movements, eddy-current artifacts, or a failed estimation of transformation parameters.

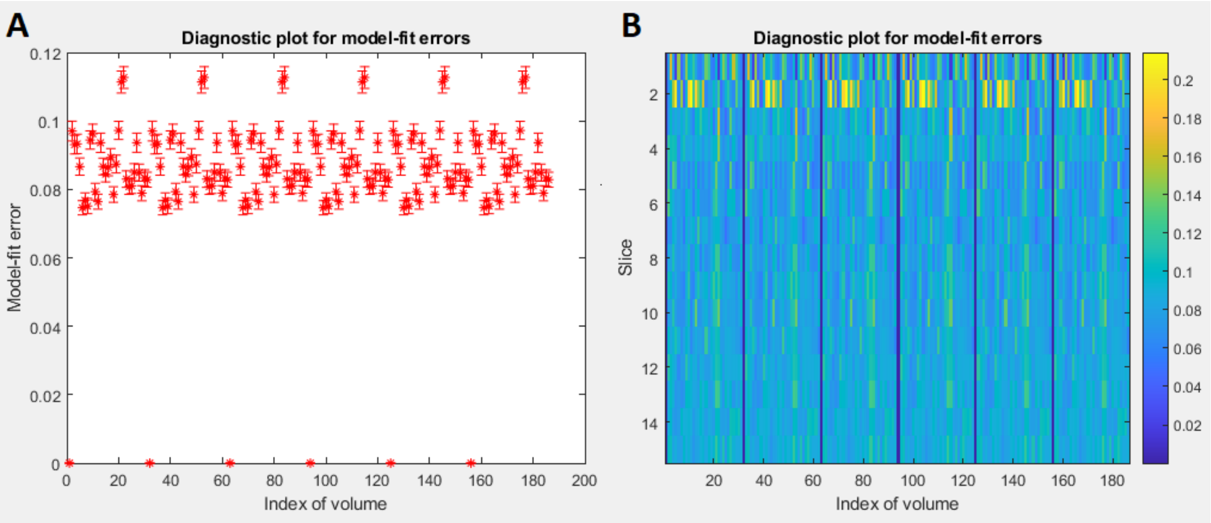

Fig. S2. Diagnostic plots, optionally generated by the *Diffusion tensor/kurtosis imaging* module, displaying the average (logarithmic) model-fit error within the provided region of interest for each volume (A) and per slice (B), demonstrated here with an in vivo spinal cord dataset and a spinal cord mask. Volumes/slices with high model-fit error (outliers) indicate a high number of corrupted volumes (e.g., due to misregistration, physiological, or other artifacts) or an inadequate model for capturing the underlying complexity of diffusion. Here, periodically occurring pairs of volumes with high model-fit errors result from an inadequate model fit due to the low signal-to-noise ratio caused by the diffusion-sensitizing gradient aligned parallel to the spinal cord. Notice that the model-fit error is highest within slice 2, which could be attributed to the presence of physiological artifacts in that location. For a more precise diagnosis of signal outliers, the user can also inspect the voxel-wise root-mean-square of the model-fit error map (suffix: RMSE-LOG_map.nii) or the 4D model-fit error map (suffix: ERROR-LOG_map.nii).

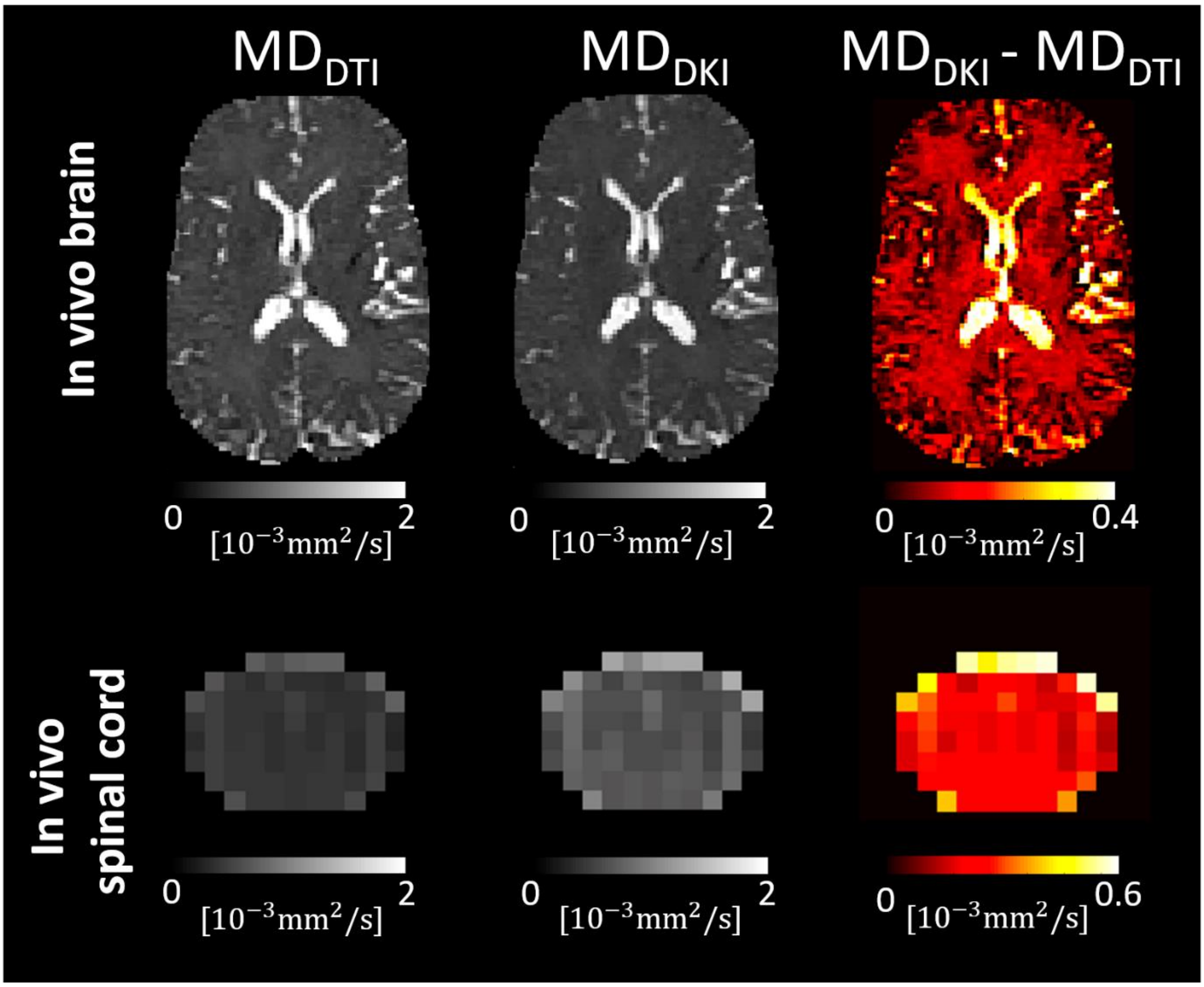

Fig. S3. Kurtosis bias in the mean diffusivity (MD) maps in an in vivo brain and in vivo spinal cord dataset (refer to Table 4 for details on the datasets). This bias, shown in the right column, refers to the difference in the estimated diffusivity values when using the lower diffusion shells only ($\mathrm{MD_{DTI}}$, tensor model, left column) or both the lower and higher diffusion shells ($\mathrm{MD_{DKI}}$, kurtosis model, middle column). On average, the kurtosis bias was 12% and 54% within the brain white matter and the whole spinal cord, respectively.

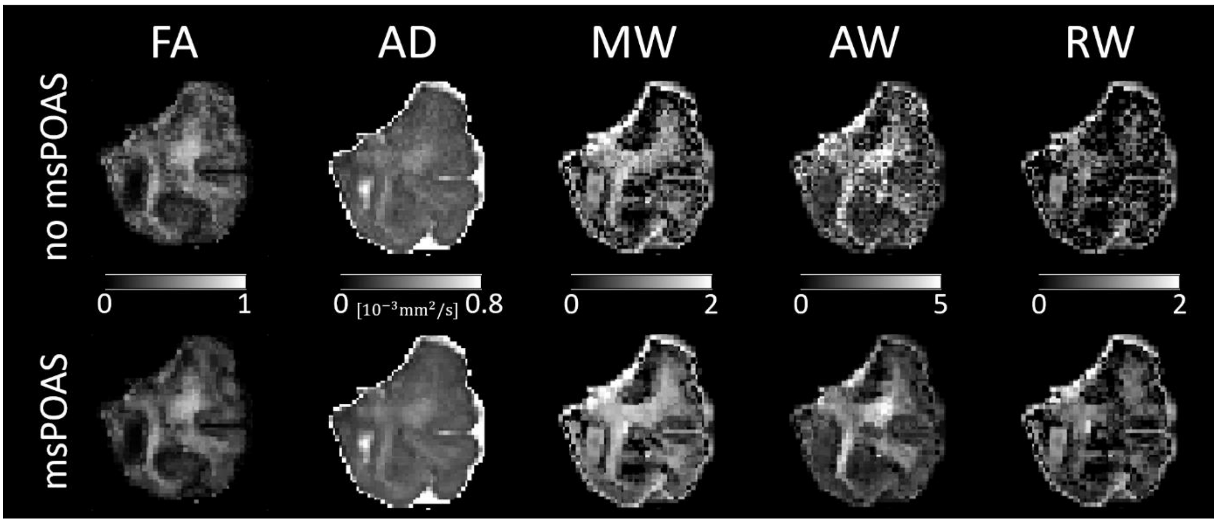

Fig. S4. Comparison of maps obtained from fitting the diffusion kurtosis model, including fractional anisotropy (FA), axial diffusivity (AD), mean kurtosis tensor (MW), axial kurtosis tensor (AW), and radial kurtosis tensor (RW) with and without applying adaptive denoising (msPOAS). The msPOAS-corrected maps appear less noisy while preserving tissue edges.

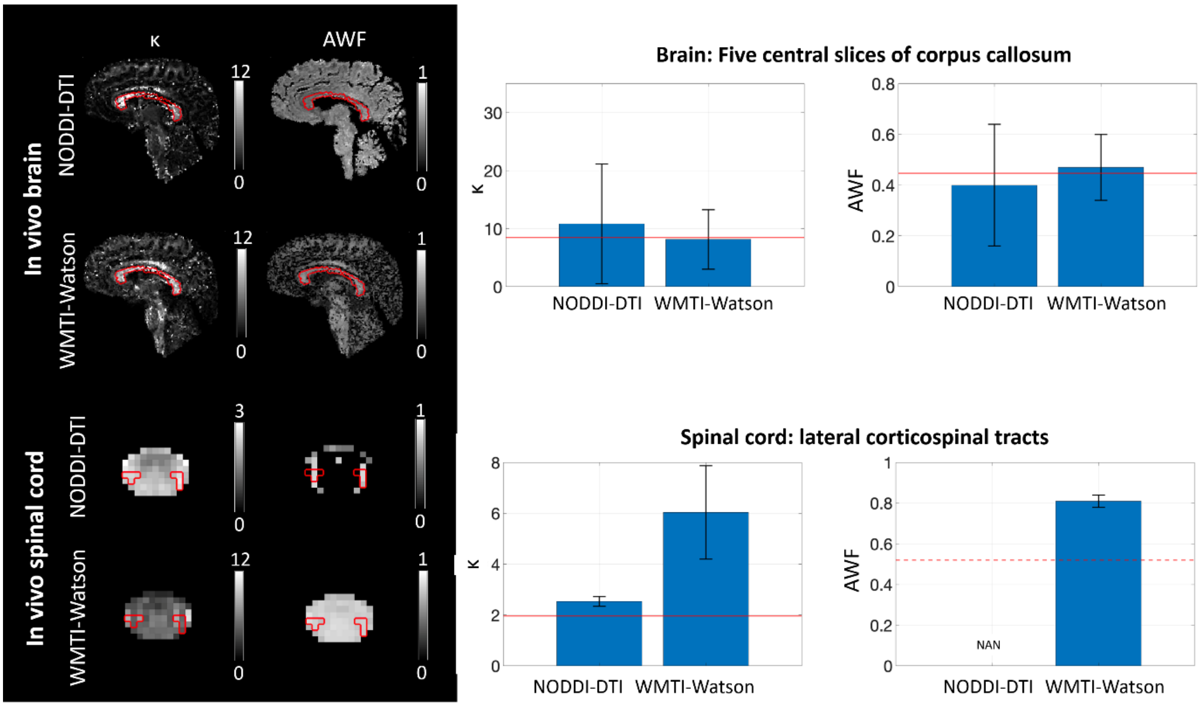

Fig. S5. Bar plots displaying the Watson concentration parameter (κ) and axonal water fraction (AWF) within the five central slices of the corpus callosum and the lateral corticospinal tracts in the spinal cord (refer to Table 4 for details on the datasets). The corpus callosum was manually segmented, while the lateral corticospinal tracts were segmented using the PAM50 spinal cord white matter atlas. The regions of interest are highlighted as red segmentation lines on the images. In the box plots, red horizontal lines represent literature values obtained from histology, while the red dotted line represents a literature value from the brain due to the absence of a corresponding value for the spinal cord. Values of orientation dispersion index reported in the literature were converted to κ using Equation (1) in Mollink et al., 2017. Within the corpus callosum, κ values were (mean ± std) 10.82 ± 10.31 and 8.14 ± 5.13 when derived from the NODDI-DTI (single shell) and WMTI-Watson model (two shells), respectively. These values fall within the range of literature values obtained post-mortem using polarized light imaging (Mollink et al., 2017). AWF values derived from NODDI-DTI (0.40 ± 0.24) and WMTI-Watson model (0.47 ± 0.13) were similar to literature values obtained using electron microscopy in a cynomolgus macaque (Stikov et al., 2015). Within the lateral corticospinal tracts, κ values derived from NODDI-DTI were notably lower than those derived from WMTI-Watson (2.53 ± 0.19 vs. 6.04 ± 1.84) and were consistent with literature values obtained in a post-mortem specimen (Grussu et al., 2017). AWF values derived from the WMTI-Watson model in the spinal cord were substantially higher (0.81 ± 0.03) compared to a literature value obtained in the brain (red dotted line). The estimation of AWF for the spinal cord was not feasible using the NODDI-DTI model, as DTI-derived mean diffusivity (MD) values fell below the range where the NODDI-DTI model provides a valid representation (refer to Equation (4) in Edwards et al., 2017). This discrepancy could be attributed to either the underestimation of MD due to kurtosis bias (Fig. S3) or the invalidity of fixed compartmental diffusivities in the NODDI-DTI model. These results indicate that WMTI-Watson yields more accurate estimation of κ and AWF for the brain, while NODDI-DTI yields a more accurate estimation of κ for the spinal cord. This could be a consequence of non-optimal b-values for kurtosis estimation in the spinal cord.

References

Edwards, L. J., Pine, K. J., Ellerbrock, I., Weiskopf, N., & Mohammadi, S. (2017). NODDI-DTI: Estimating neurite orientation and dispersion parameters from a diffusion tensor in healthy white matter. *Frontiers in Neuroscience*, *11*, 720. https://doi.org/10.3389/fnins.2017.00720

Grussu, F., Schneider, T., Tur, C., Yates, R. L., Tachrount, M., Ianuş, A., Yiannakas, M. C., Newcombe, J., Zhang, H., Alexander, D. C., DeLuca, G. C., & Gandini Wheeler-Kingshott, C. A. M. (2017). Neurite dispersion: a new marker of multiple sclerosis spinal cord pathology? *Annals of Clinical and Translational Neurology*, *4*(9), 663–679. https://doi.org/10.1002/acn3.445

Mollink, J., Kleinnijenhuis, M., van Cappellen van Walsum, A.-M., Sotiropoulos, S. N., Cottaar, M., Mirfin, C., Heinrich, M. P., Jenkinson, M., Pallebage-Gamarallage, M., Ansorge, O., Jbabdi, S., & Miller, K. L. (2017). Evaluating fibre orientation dispersion in white matter: Comparison of diffusion MRI, histology and polarized light imaging. *NeuroImage*, *157*, 561–574. https://doi.org/10.1016/j.neuroimage.2017.06.001

Stikov, N., Campbell, J. S. W., Stroh, T., Lavelée, M., Frey, S., Novek, J., Nuara, S., Ho, M. K., Bedell, B. J., Dougherty, R. F., Leppert, I. R., Boudreau, M., Narayanan, S., Duval, T., Cohen-Adad, J., Picard, P. A., Gasecka, A., Côté, D., & Pike, G. B. (2015). In vivo histology of the myelin g-ratio with magnetic resonance imaging. *NeuroImage*, *118*, 397–405. https://doi.org/10.1016/j.neuroimage.2015.05.023
